# Health service planning contributes to policy dialogue around strengthening district health systems: an example from DR Congo 2008–2013

**DOI:** 10.1186/s12913-014-0522-4

**Published:** 2014-10-31

**Authors:** Dheepa Rajan, Hyppolite Kalambay, Mathias Mossoko, Dieudonné Kwete, Joseph Bulakali, Jean-Pierre Lokonga, Denis Porignon, Gerard Schmets

**Affiliations:** Department for Health Systems Governance and Financing, World Health Organization, 20 Avenue Appia, Geneva, 1202 Switzerland; Directorate of Planning, Ministry of Health, Blvd du 30 juin, Kinshasa, Congo; Directorate of Primary Health Care Development, Ministry of Health, Blvd du 30 juin, Kinshasa, DR, Congo; Prime Minister’s Office, Avenue De Lemera, Kinshasa, DR, Congo; Global Fund Country Coordination Mechanism Secretariat, Ministry of Health, Kinshasa, DR, Congo; World Health Organization, Boîte postale 1899, Kinshasa, DR, Congo

## Abstract

**Background:**

This case study from DR Congo demonstrates how rational operational planning based on a health systems strengthening strategy (HSSS) can contribute to policy dialogue over several years. It explores the operationalization of a national strategy at district level by elucidating a normative model district resource plan which details the resources and costs of providing an essential health services package at district level. This paper then points to concrete examples of how the results of this exercise were used for Ministry of Health (MoH) decision-making over a time period of 5 years.

**Methods:**

DR Congo’s HSSS and its accompanying essential health services package were taken as a base to construct a normative model health district comprising of 10 Health Centres (HC) and 1 District Hospital (DH). The normative model health district represents a standard set by the Ministry of Health for providing essential primary health care services.

**Results:**

The minimum operating budget necessary to run a normative model health district is $17.91 per inhabitant per year, of which $11.86 is for the district hospital and $6.05 for the health centre. The Ministry of Health has employed the results of this exercise in 4 principal ways: 1.Advocacy and negotiation instrument; 2. Instrument to align donors; 3. Field planning; 4. Costing database to extract data from when necessary.

**Conclusions:**

The above results have been key in the policy dialogue on affordability of the essential health services package in DR Congo. It has allowed the MoH to provide transparent information on financing needs around the HSSS; it continues to help the MoH negotiate with the Ministry of Finance and bring partner support behind the HSSS.

## Background

This paper, a resource planning exercise done as an integral part of the Democratic Republic of Congo (DR Congo) Ministry of Health’s core functions, demonstrates how rational operational planning based on a health systems strengthening strategy can greatly contribute to health policy dialogue and decision-making over several years. It explores the modalities of operationalizing a national strategy at district level by elucidating a normative model district resource plan which details the resources and associated cost implications of providing a comprehensive essential health services package at district level.

Published material on health service planning on a normative and comprehensive basis for a full health district is rare. There exist several examples and publications on health service planning for a single facility, usually hospitals, or for a single program (often HIV) with the aim of estimating future resource need [1]. What makes DR Congo’s resource planning unique is the fact that the full essential health services package was planned for (over 200 interventions), including district management and health prevention and promotion activities; in addition, it was done on a normative basis within the framework of the national health sector strategy (HSSS) so as to assure a certain service quality level while taking into consideration the local context.

We demonstrate in this descriptive analytical study relying on primary and secondary data that the normative model district is a realistic model DR Congo district, with standardized functionality, resource usage, utilization, and interventions provided. This paper also gives examples of the longer-term impact of this work on the DRC health system.

### DR Congo’s health system

DR Congo’s health sector faces many challenges, including high maternal and infant mortality [2,3], poor infrastructure [4], security risks, and a growing burden of non-communicable diseases [5]. The health system was severely destroyed by civil war (1997–2003). DR Congo’s MoH was thus faced with a daunting challenge following the 2006 democratic elections. Fortunately, the country had a long tradition of primary health care-based district health systems; the MoH decided that a rapid rehabilitation of these district health facilities was necessary to more adequately meet the demand brought on by the post-conflict challenges. In order to be able to operationalize destroyed or poorly functioning district health facilities, DR Congo MoH recognized the need to:

(i) outline a comprehensive plan for strengthening districts
(ii) identify funds to implement the plan

(i) *outline a comprehensive plan detailing the modalities for strengthening health districts*
  In 2006, DR Congo released its Health Systems Strengthening Strategy (HSSS) [6]. The HSSS spells out the backbone of the Congolese health system to be *the decentralized health district* which integrates primary care and first-referral services, represented respectively by the HSSS *Minimum and Complementary Package of Activities* (together, the MPA and CPA represent the essential health services package for district level, covering the health centre and district hospital respectively), under one administrative structure for health service delivery. The HSSS was subsequently endorsed by bilateral and multilateral partners and marked an important step in regaining confidence lost by Congolese citizens in their public health system [7].
  This decentralized HSSS health district is an integrated health system with 2 levels: a network of health centres and a 1^st^ level referral, or district hospital. Its primary concern is the health needs of the population covered. In this model, the health centre network’s activities are complementary to the district hospital’s activities, with little overlap, and backed by a functioning referral system.
(ii) *identify funds to implement the plan*
  Soon after the adoption of the HSSS, the MoH realized that it would be difficult to mobilize development partners to concretize and fund the strategy without an estimation of the necessary resources and operational budget. A resource planning team was then created in 2007 within the Planning Directorate to study the resource implications at health district level for delivery of the *Minimum and Complementary Package of Activities* (*MPA and CPA*).

## Methods

The aim of the resource planning team in DR Congo was to operationalize the Health System Strengthening Strategy (HSSS) at district level, specifically:

- To inform the policy dialogue on affordability of the HSSS essential health services package to be provided at district level
- To provide an absolute minimum resource base and operational budget for the HSSS essential health services package to be provided at district level, which can be used as a normative model across districts
- To build capacity and establish sustainability of resource planning activities within the DR Congo Ministry of Health by ensuring ownership of the resource planning methodology

**Figure 1:**
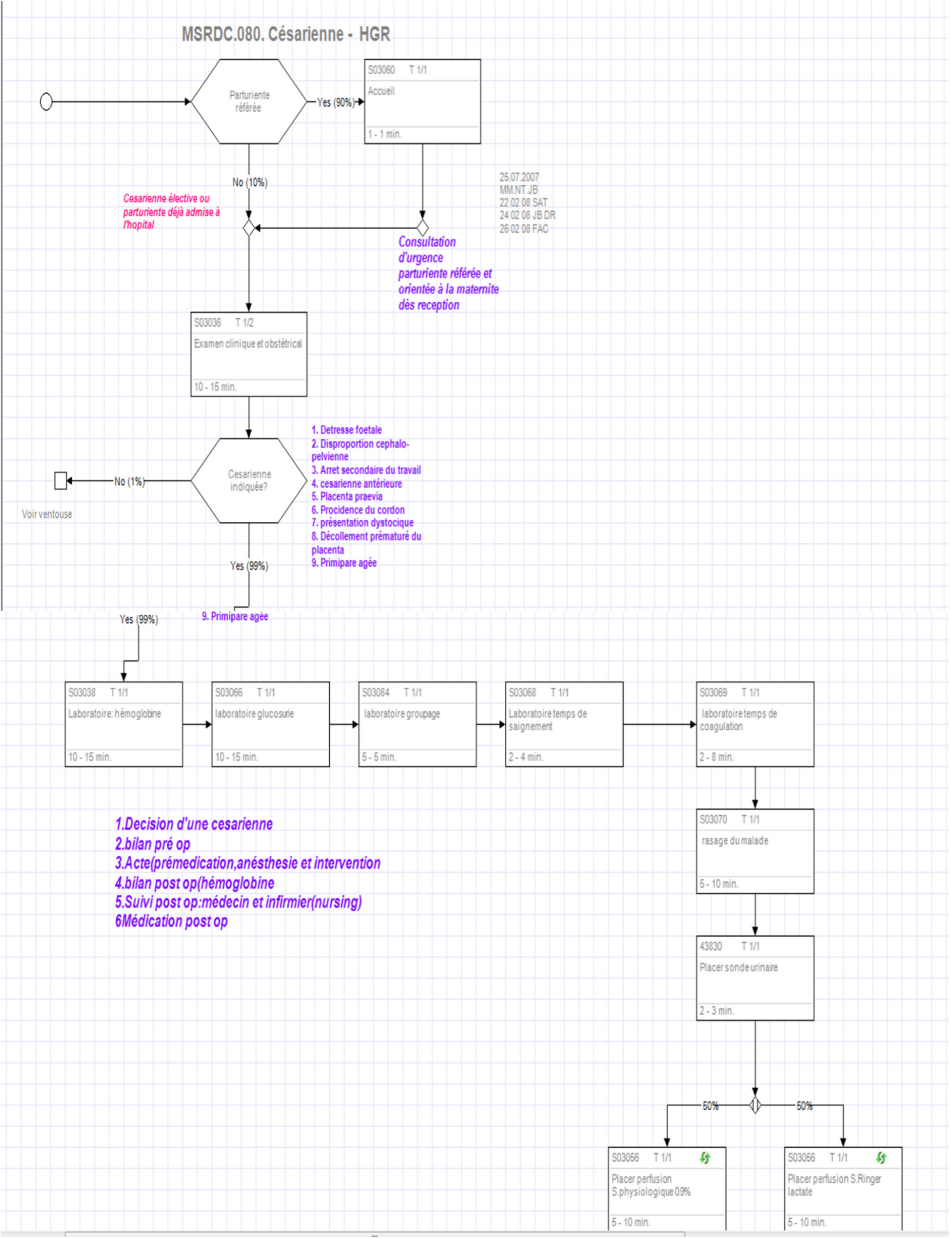
**An example iHTP intervention algorithm: caesarian section.** The case load for Caesarian section is 41 patients per year at a normative model district hospital (see below for methodology on determination of case load/utilization rate). The human resources necessary to perform this intervention are 1cashier, 1 usher, 1 A1 nurse, 1 A2 nurse, 1 doctor, 1 A1 lab technician, 1 A2 lab technician, and an anesthetist. Similar lists are available per intervention for equipment, medical devices, supplies, pharmaceuticals, and infrastructure.

To address its objectives, the MoH decided to use WHO’s integrated Healthcare Technology Package (iHTP) method. This planning model and software-based tool calculates the mix of needed inputs to deliver a defined number of selected clinical (for example, Cesarean section) and non-clinical (for example, supervision by district health authorities of health centres) interventions belonging to the MPA and CPA. Each single intervention (clinical; or non-clinical, for eg., health promotion activities) is broken down into procedures (ex: ‘examination of patient’, ‘measure blood pressure’, ‘prepare for outreach activities’) in algorithm format (see example below). Each procedure is then broken down into its resources, i.e., human resource, medical devices, equipment, drugs, and facility necessary for that particular procedure. Each intervention is thus made up of several procedures; with over 200 total interventions, the database created in DRC contains well over 2000 procedures. Prices and salaries for each resource are then entered into the software and costs were calculated as an operating cost assuming that basic capital equipment and the building itself are standing and can be used. Large medical equipment depreciation cost was calculated according to the actual duration of time it was used per procedure across a defined equipment-specific life cycle (usually 5–20 years). Facility depreciation cost was also calculated according to usage per procedure across a defined facility building cost. Building maintenance, utilities, and cleaning costs were added to total facility operating costs. Consumable items and drug quantities and their associated costs were calculated according to the total amount necessary for each procedure within each intervention. Figure 1 illustrates an example iHTP intervention algorithm.

It is important to note that capital investment costs necessary to rehabilitate health facilities, pre-service human resource training, and non-health facility infrastructure were not included in this resource planning and costing exercise. Table 1 summarizes principal costs which were included and not included.

**Table 1:** **Principal types of costs of the exercise**

**Costs which were included in this exercise**	**Example**	**Costs which were not included in this exercise**	**Example**
Costs of clinical intervention	Cost of procedures such as general anaesthesia, applying a cast, or X-ray	Capital investments necessary to rehabilitate health facilities	Repair of facility building or building a new one
Referral costs as defined by country	Cost of ambulance and medical services during transport	Pre-service human resource training	University education for Medical Officers
Health facility administrative costs	Cost of hospital manager and office equipment	Infrastructure outside the health facilities	Paved roads for better health facility access
District health administration	Cost of district health manager and supervisory visits		

The aim of this particular resource planning exercise in DR Congo was to answer the following question: how much is necessary per inhabitant and per year to operationally run a health centre or district hospital, assuming that the basic infrastructure exists and that the personnel provide quality services as per the standards in DR Congo?

### Normative model health district

The normative model Health Centre (HC) and normative model District Hospital (DH) represent a well-functioning health district with the absolute minimum resource base necessary to function and thereby, a standard set by the Ministry of Health for providing essential primary health care services for all district inhabitants. The normative model HC and DH were designed based on the *Minimum and Complementary Health Packages* respectively, as elucidated in the HSSS.

A health district is defined in the HSSS as a zone covering 100 000 inhabitants, with one district hospital who takes almost exclusively referred cases from a network of 10 health centres. A district management team is based at the district hospital and supports, manages, and supervises district health activities. The detailed list of interventions for both the MPA and CPA as well as the resource needs for all procedures contained in each health package intervention were elaborated together with local senior clinicians and health authorities.

Health service utilization rates were linked to each intervention. Health facility registries across well-functioning health centres and 1^st^ referral hospitals (district hospitals) were studied to infer a normative average utilization rate for the normative health centre and the normative district hospital. The aim here was to get a real sense of the number of cases a normalized HSSS-based health facility would be charged with for each single intervention in a local DR Congo context. Following this method, the normative model utilization rate was estimated to be 0.4 cases/inhabitant/year for the health centre level and 0.15 for the district hospital level.

Normative district hospital staff salaries were based on the Mbudi agreement, which fixes an agreed minimum salary for public employees and was signed between the government and the public services union [8]. Normative medical devices, equipment, drugs, and infrastructure data entered into the iHTP health service algorithms were based on national clinical guidelines, textbooks used in local medical schools, and expert consensus opinion.

## Results and discussion

In this section, we first present the results of the iHTP calculated costs for the normative model health centre and the normative model 1^st^ level referral hospital. We then summarize the overall total costs for the whole normative model health district and describe what this cost includes. Finally, we present practical benefits and results of the resource planning work in DR Congo (Table 2).

**Table 2:** **Summary table**: **health district model normative costs**

	**Health centre**	**1st level referral hospital**	**Total**
Operating budget/inhabitant/year	$6.05	$11.86	$17.91
Utilization rate (cases/inhabitant/year)	0.4	0.15	N/A
No. of activities included	98	117	215

### Health centre

At a utilization rate of 0.4 cases per inhabitant per year, with 98 curative and preventive interventions (for example, appendicitis or antenatal care), reflecting the full HSSS-defined *Minimum Package of Activities*, the health centre operating budget necessary to cover required resources is $6.05/inhabitant/year.

These resources include 2 nurses, a lab technician, a receptionist, and a maintenance agent, representing almost half (45%) of the total operating budget. Almost 1/4 (22%) of the total health centre operating budget is needed for disposable supplies. Another 1/4 (26%) is necessary for facility maintenance, depreciation, and utilities. Only 7% goes for drug supplies and a negligible <1% is necessary for reusable equipment usage.

### 1^st^ level referral hospital

At a utilization rate of 0.15 per inhabitant per year, with 117 curative, preventive, managerial, and supervisory activities, reflecting the full HSSS-defined *Complementary Package of Activities*, the 1^st^ level referral (district) hospital operating budget necessary to cover required resources is $11.86/inhabitant/year.

This operating budget includes 21 nurses, 5 doctors, 1 nurse-anaesthetist, 4 lab technicians, 1 X-ray technician, 1 physiotherapist, 1 pharmacist, 1 nutritionist, and 6 administrative, facility maintenance, and management personnel. Thirty-five percent of the total operating budget is necessary to cover human resource salaries. Approximately 1/4 of the total operating budget is required for pharmaceuticals, with the top five drugs being amoxicillin, serum Glucose 5%, Pentamidine isethionate, Nelfinavir, and Abacavir. Twenty-one percent of the operating budget is necessary for disposable items and 16% for facility usage (depreciation, maintenance, and utilities). Less than 1% of the operating budget is used for reusable medical equipment.

### Normative model health district

The operational budget cost for a full-fledged normative HSSS health district, with 10 health centres and 1 district hospital, is thus $17.91/inhabitant/year. This HSSS health district offers a comprehensive essential health services package (*Minimum* and *Complementary Package of Activities*) including maternal, neonatal, and child health care; reproductive health services; surgery care; internal medicine and chronic care; tuberculosis, HIV/AIDS, and malaria care; communicable disease care; preventive medical services; management and maintenance services; etc. The HSSS health district is assumed to have a functioning referral system, that is, there is little overlap of health services between the health centre and the district hospital level.

Practical benefit of the resource planning work (grouped according to objectives of this exercise):

#### Exercise objective 1: To inform the policy dialogue on affordability of the HSSS essential health services package to be provided at district level

The Ministry of Health employed the normative HSSS district resource planning results in 4 ways linked to the policy dialogue on affordability of the HSSS:

1. *Advocacy and negotiation instrument*
2. *Instrument to align donors*
3. *Field planning*
4. *Costing database*

1. *Advocacy and negotiation instrument*: *to advocate and negotiate for more funding for the* (*decentralized*) *health districts*
  Negotiations with the Ministry of Finance were better informed by the DR Congo costing database. The government health budget was elaborated based on the normative model health district and the costing results obtained from this exercise. In the past, costing and budgeting were usually based on the previous year’s estimations, without an updated analysis of health sector needs.
  DR Congo submitted a successful proposal for the *Global Fund* - *Health Systems Strengthening window*, *9*^*th*^*Round*. The normative model health district planning results served as a point of reference to propose channeling 9^th^ Round funds to the health district as a unit as opposed to specific projects or programs. This was possible because the planning and costing work itself was done for the *district level*, integrating all services at that level, including management and prevention.
  The comprehensive resource budget for a normative model district allowed the MoH to better negotiate with donors based on transparent information: by demonstrating a solid list of interventions, resources, and associated budget needs for a defined health package, the MoH found donors much more willing to consider country planning interests.
2. *Instrument to align donors*: *to channel existing resources towards the health district level*
  Large proportions of donor funds in the DR Congo have traditionally gone to management and administration of programs -- for example, up to 40% of the *European Development Fund* (*EDF*)’*s 9*^*th*^*Program for Health* in DR Congo was spent on management and administration [9]. By demonstrating that the actual budget needs for operating a well-functioning health district based on the HSSS in DR Congo was higher than donors had planned for, the MoH was able to make the case that more funds need to be channeled to the field, i.e., to district health level, rather than into administration and overhead.
  The *European Development Fund* (*EDF*)’*s 10*^*th*^*Program for Health* in DR Congo was planned and drafted with the inclusion of the MoH resource planning team for key planning and budgeting activities [10]. The budget of the *10*^*th*^*Program for Health* is €51 million; the initial EDF plan was to cover 100 health districts with this amount. The MoH strongly advocated for and successfully negotiated a reduction in the number of districts covered to 20–25, based on the results of the normative model health district planning exercise which demonstrated the need for a total operating budget of at least $17.91/inhabitant/year to adequately run a health district providing good quality services.
  DR Congo drafted a new national health plan in 2011 (*Plan national du développement sanitaire* 2011–2015), which is aligned to the Poverty Reduction Strategy Paper (PRSP). One of the principal focus areas of the new plan is to support and strengthen the health district. The MoH’s discussions with international partners on the affordability and budget accorded to districts were based on the normative health district resource planning results. The Medium-Term Expenditure Framework (MTEF) for the new health plan drew heavily from the costing database.
3. *Field planning*: *to assist MoH*, *NGOs and donors in the provinces* (*field*) *to plan their district health services*
  In 2009/10, in order to better assess health facility needs in districts, the MoH resource planning team collaborated with an international NGO in 2 pilot districts by calculating a detailed resource base and budget needs for current actual health service provision and quality; they analyzed the gap between the current, low-quality ‘reality’ scenario and the normative model health district target of the MoH. This collaborative work resulted in an updated equipment inventory in these 2 districts, budget provisions for a better supply of drugs and consumable items, and a debate on a new human resource plan. For the MoH, this collaboration with a development partner has demonstrated a practical example of normalizing health service provision and quality across districts which have varying levels of service quality, donor support, and history.
  In 2013, the same exercise as presented above was undertaken in 2 health centres and 1 district hospital in another province by a bilateral agency, in close collaboration with the MoH and its resource planning team. This exercise is currently on-going but attests to the usefulness of doing a gap assessment based on the reality in health facilities vis à vis a modelled and costed norm.
  An operating budget simulation was done for the newly-built Hôpital du Cinquentenaire, a national level tertiary care specialty hospital on the basis of the iHTP simulations done at the district hospital level. This budget forecast assisted in having a more concrete idea of the level of funds which need to be included in the Ministry of Health budget in order to run this specialized health facility.
4. *Costing database*
  A national-level assessment conducted in 2012 studied the required type, amount, and costs of medicines and health supplies for operationalization of the new national health plan (PNDS) [11]. The costing database was used to conduct a separate sub-analysis of medicines and supplies and this was supplemented with additional data linked to the specific terms of reference of the assessment. The continued use of the costing database years after it was originally created demonstrates the high utility value of such data sets.
  The district-level resource planning work is serving to feed into the cost analysis of the National Health Plan (*Plan National du Developpement Sanitaire*) by providing input into the more strategic OneHealth [12] costing exercise currently on-going in 2013.

#### Exercise objective 2: To provide an absolute minimum resource base and operational budget for the HSSS essential health services package to be provided at district level, which can be used as a normative model across districts

Based on these results, the normative model health district costs can be considered as an absolute minimum cost base for the essential health services package. A similar type of normative planning exercise was undertaken by a bilateral agency in one of the districts under their responsibility which was known to function well (Kenge) [13]. Their results at health centre level was $4.20/inhabitant/year and at hospital level $10.20/inhabitant/year. Since these numbers did not include health district management and HIV, one can state that these numbers correspond very well to the MoH’s normative model basic health centre and district hospital costs. The bilateral agency exercise was done with a similar premise (normative) and context and thus at least partly validates, by comparison, the MoH’s results.

Congolese health districts are financed by a wide variety of donors, leading to varying services and utilization rates across districts. This resource planning exercise being HSSS-based and normative in nature, allows for a common resource base standard at which to aim across districts, so that bridging the financing gap becomes easier and more equitable. It thus serves as a realistic model to strive for in terms of functionality, resource usage, utilization, and interventions provided in a fully functioning DR Congo health district.

#### Exercise objective 3: To build capacity and establish sustainability of resource planning activities within the DR Congo Ministry of Health by ensuring ownership of the resource planning methodology

The Planning Directorate, MoH, invested heavily in building resource planning capacity. Three experts worked on the iHTP resource planning and costing database over 9 months to build up the original database with the *MPA* and *CPA* for the health district. This resource planning team also supported district management teams in district health planning, and collaborated with provincial and district health authorities in formulating their provincial and district health plans.

The resource planning team has thus fully adapted and embedded the global instrument of iHTP to the local DR Congo context via the planning and costing work for the HSSS normative district. The effort made to adapt the tool included WHO training and support to the MoH team for one year, corresponding to approximately 300 man-hours from international staff. The continued existence and deployment to the districts of the resource planning team is testimony to the MoH’s ownership of the iHTP resource planning methodology as well as the country’s long-term strategy to maintain planning capacity within its institutions.

Limitations to the study include the lack of available and reliable data, especially concerning utilization rates, case load, and other facility-based data. Another limitation was the fact that the iHTP tool was used to model non-clinical interventions (management of the district, for example) for which it was not initially conceived. The authors of this paper were conscious of these limitations and made best-guess estimations based on available data and mapped out iHTP non-clinical algorithms in a way which was most compatible with the tool structure.

## Conclusions

The above results have been key in the policy dialogue process in DR Congo over the last few years: they have supported the Ministry of Health in the national discussion (policy dialogue) on affordability of the *Minimum* and *Complementary Packages of Activities* of the HSSS by providing an absolute minimum possible resource base and associated yearly required operational budget for the MPA and CPA. The resource planning work has allowed the MoH to provide information in a transparent and rational manner on financing needs around the HSSS; it has helped and continues to help the MoH negotiate with the Ministry of Finance and bring national and international partners behind the HSSS concretely and operationally, following their political commitment. In addition, it has contributed to the standardization of health service provision and quality across districts in a country with a fragmented donor setting.

When comparing this study’s results with the international literature, we find that in Kenya, a 2011 study [14] on the essential health services package calculated the per capita cost at 18.65 EUR with 37% of the cost going to salaries and 22% for drugs and medical supplies. Even though the setting and the costing methodology used (a step-down allocation method using cost centres) were quite different from ours (bottom-up methodology), the results are surprisingly similar. The main difference is the considerably larger drugs and supplies component in the DR Congo 1^st^ level referral hospital compared to the Kenyan one. This could be due to the Kenyan costs being based on the drugs which the hospitals dispense as well as drugs which the patients purchase on their own when the hospital has a stock-out. Patients may not be able to afford to purchase all the drugs which they could and should, thus underestimating drug costs. In the DR Congo study, the drug costs were based on the iHTP intervention algorithms which were normative in nature, calculating the total drugs which the patient *should* take.

A 2004 study by the same author on hospital services in Vietnam [15] found that a higher proportion of costs from district hospitals were going for personnel (61% and 64% in the 2 district-level hospitals studied). The district hospital drugs and supplies component was 25% and 24% respectively. The per capita cost of the services offered at district hospital is difficult to compare with our study as the catchment area for the district hospitals in Vietnam have a very broad range (from 50 000 to 300 000) and the exact type and nature of the health package is not elaborated upon. The higher personnel cost proportion could be due to higher salaries in Vietnam; the lower hospital drugs and supplies cost could be linked to the fact that actual costs were studied and not the normative cost of what health services should cost according to a defined clinical pathway, such as in our DR Congo study.

The same holds true with respect to an older study by Mills et al. [16] in Malawi which looked specifically at district hospital costing. The distribution of costs demonstrated 27-39% for salaries and 24-37% for medical supplies and drugs. Again, the salary proportion mirrors our DR Congo study, but the medical supplies and drugs at district hospital level was considerably higher in DR Congo – again, most likely due to the difference between evaluating actual costs of services provided vs. evaluating normative model costs based on intervention algorithms.

In DR Congo, the process of determining the normative HSSS health district resource base served to build in-house MoH capacity in resource planning and ensured its establishment within the Planning Directorate. This has led to a true ownership of the resource planning methodology as well a real increase in planning capacity in the MoH.

The results of this comprehensive, normative, HSSS-based resource planning work were effectively used by policy-makers for purposes of advocacy; to align donors to the HSSS at district level; and for field district planning. Donors, implementation agencies, district and provincial health authorities were politically aligned to the HSSS from the beginning; however, a transparent and comprehensive resource plan allows them to be operationally and functionally aligned as well. Those working at district level are now integrating the MoH’s normative model health district resource plan into their own district planning activities -- at least in a few initial districts at the moment -- by adapting the resource planning methodology to their local district epidemiology, utilization rates, and resource base, with support from the MoH.

The amount donors and government are currently spending in the health sector per capita in DR Congo (approximately $6-$7/inhabitant/year) is simply too low to enable districts to offer the services outlined by the HSSS. At the moment, households spend high amounts ($4.50/inhabitant/year) [17] and this is still insufficient to reach the level of the normative HSSS-based district. At least $6.41/inhabitant/year is needed to bridge the gap in a country with 75 million inhabitants. Additional financing from both internal and external sources will therefore be necessary in order to provide high quality integrated care with the ultimate aim of universal health coverage. With a competent and flexible resource planning team at the MoH supporting districts in their district health plans, additional financing can be best channeled to where it is needed most. Comprehensive primary health care services in a decentralized health district is not cheap but can reap the benefit in improved health service uptake -- a decisive step towards universal health coverage -- and ultimately improved health outcome for the population [18].

## Abbreviations

DH: District hospital
EDF: European Development Fund
HC: Health Centre
HSSS: Health System Strengthening Strategy
HIV: Human Immunodeficiency Virus
iHTP: integrated Healthcare Technology Package
MoH: Ministry of Health
MTEF: Medium-Term Expenditure Framework
NGO: Non-governemental Organization
PNDS: *Plan National du Développement Sanitaire*, or, National Health Development Plan
PRSP: Poverty Reduction Strategy Paper
